# Dynamic changes in the endophytic bacterial communities of huanglongbing-affected periwinkle leaves

**DOI:** 10.3389/fmicb.2025.1607062

**Published:** 2025-11-27

**Authors:** Xiaofei Liu, Yuli Dai, Lin Gan, Chengzhong Lan, Hongchun Ruan, Lizhen Zheng, Yu Zheng, Yun Gan, Xiujuan Yang

**Affiliations:** Fujian Key Laboratory for Monitoring and Integrated Management of Crop Pests, Institute of Plant Protection, Fujian Academy of Agricultural Sciences, Fuzhou, China

**Keywords:** huanglongbing, periwinkle leaves, endophytic bacteria, dynamic changes, high-throughput sequencing

## Abstract

The pathogenetic mechanism of citrus huanglongbing (HLB) disease caused by *Candidatus Liberibacter asiaticus* (Clas) remains unclear, particularly its association with host-related microbial communities. It has been demonstrated that HLB infection alters endophytic bacteria, which may be the cause of disease initiation. In this study, periwinkle plants were used as a model and uniformly inoculated by grafting with HLB-infected citrus shoots. Leaf samples were collected at 30, 60, and 90 days post-inoculation (dpi) to assess alterations in endophytic bacterial communities using high-throughput sequencing, and uninfected periwinkle plants served as controls. Across all samples, 24 bacterial phyla and 300 genera were identified. In healthy plants, the endophytic bacterial diversity and richness remained relatively stable over time, with *Arcobacter*, *Pseudomonas*, and *Giesbergeria* identified as the predominant genera. In contrast, HLB-infected plants exhibited a marked decline in bacterial diversity over time, though richness was not significantly affected. Notably, the relative abundance of *Candidatus Liberibacter* increased significantly, and it became the dominant genus by 30 dpi, followed by *Arcobacter* and *Pseudomonas*. HLB infection was also associated with a sustained reduction in abundance of certain genera, including *Anaerovorax*, *Neisseria*, and *Paeniclostridium*. This study provides a detailed overview of the temporal dynamics of leaf endophytic bacterial communities following HLB infection of periwinkle plants. This study enhances our understanding of host–microbe–pathogen interactions and may guide future studies aiming to select useful microbial candidates for HLB alleviation and plant resilience.

## 1 Introduction

Citrus huanglongbing (HLB), also known as citrus greening, is a devastating disease widespread in Asia, Africa, and the Americas (Bové, 2006; Ma et al., 2022). An HLB infection causes severe pathological effects in plants, including sieve pore blockage, phloem dysfunction, and the disruption of chloroplast grana structures (Ma et al., 2022). Affected trees display characteristic symptoms such as blotchy leaf mottling, premature flowering, and small, bitter fruits, often leading to death within a few years (Thakuria et al., 2023). Notably, citrus trees infected with HLB can remain asymptomatic for months to several years postinfection (Pandey et al., 2022). During the asymptomatic period, the pathogen can be transmitted via psyllid vectors, leading to orchard-wide dissemination and significant economic losses (Thakuria et al., 2023; Zhao et al., 2025).

Currently, no HLB-resistant citrus varieties have been identified (Zhao et al., 2025), and there is no effective cure for this disease. Primary HLB management strategies focus on controlling psyllid population and removing infected trees. However, these measures are insufficient for long-term HLB mitigation, particularly considering the asymptomatic phase of infection in citrus trees (Dominguez et al., 2023). The causal pathogen of HLB, *Candidatus Liberibacter* spp., is a phloem-limited, gram-negative *α*-proteobacterium that cannot be cultured to fulfill Koch’s postulates (Merfa et al., 2019; Pandey et al., 2022). Three species of *Candidatus Liberibacter* have been characterized: *Candidatus Liberibacter asiaticus* (CLas), *Candidatus Liberibacter africanus* (CLaf), and *Candidatus Liberibacter americanus* (CLam). Of these, CLas is prevalent in Asia (Thakuria et al., 2023).

To gain a better understanding of this pathogen, recent studies have investigated responses of the endosphere when infected, with emphasis on changes in the microbial diversity in endophytes (Dominguez et al., 2023). The interaction between the pathogen and other endophytes can be linked to the failure to culture the bacterium *in vitro* (Bové, 2006; Li et al., 2012). Therefore, an endophytic community study would help provide potential biocontrol agents for HLB (Sechler et al., 2009; Thakuria et al., 2023).

Endophytes are microorganisms that inhabit plant tissues and do not cause any morphological modifications, and they usually benefit the host plants (Dominguez et al., 2023). Serving as mutualists or commensals, some of the endophytes possess the ability to enhance host tolerance to biotic and abiotic stresses (Yang et al., 2025b). Surprisingly, some compounds derived from endophytes are capable of eliciting plant defense responses against diseases. These compounds have the potential to support the development of biocontrol strategies against HLB and other plant diseases (Dominguez et al., 2023). However, there are limited comparative studies on endophytic bacteria colonizing healthy and HLB-affected citrus plants under natural field conditions (Sagaram et al., 2009; Wang et al., 2010; Wang et al., 2014; Xiong et al., 2017). Moreover, field-based research is often complicated by factors such as variability in infection duration, individual plant differences, and environmental heterogeneity (Shen et al., 2013; Harrison and Griffin, 2020). In addition, the extended latency period of symptom development in citrus trees makes timely experimentation challenging.

To address these limitations, Madagascar periwinkle (*Catharanthus roseus*) has been recognized as an ideal non-host model system. It exhibits symptoms similar to the citrus disease when infected with citrus HLB-causing bacteria via dodder (*Cuscuta campestris*) (Pandey et al., 2022). Periwinkle possesses several experimental advantages such as the rapid development of *Candidatus Liberibacter* spp., growth within controlled greenhouse conditions, and faster symptom expression (Villechanoux et al., 1992; Sagaram et al., 2009; Pandey et al., 2022). Although many studies have investigated microbial processes in the phyllosphere and rhizosphere, functional responses of endophytic bacterial communities to invading pathogens have mostly not been explored yet. Specifically, few studies have investigated the temporal dynamics of endophytic bacterial diversity following HLB development. Understanding these alterations is pivotal to deconstructing the process of HLB pathogenesis.

Based on previous studies, we hypothesized that HLB infection would gradually alter the diversity and structure of the endophytic bacterial community in periwinkle leaves and that the relative abundance of the HLB pathogen would correlate either positively or negatively with specific endophytic bacterial taxa. Therefore, the objectives of this study were as follows: (1) to compare the diversity and community structure of leaf endophytic bacteria in healthy and HLB-infected periwinkle plants and (2) to assess temporal changes in these communities, as well as the abundance of the HLB-associated bacterium during infection. Our study revealed notable shifts in both the diversity and composition of endophytic bacteria between infected and healthy plants, as well as among different time points following infection.

## 2 Materials and methods

### 2.1 Sample collection

The CLas strain used in this study was generously provided by Dr. Chong Ke from the Pomology Research Institute at the Fujian Academy of Agricultural Sciences, Fuzhou, China. This strain was initially isolated from Guanximiyou pomelo plants and subsequently transmitted to periwinkle plants via dodder. HLB-symptomatic periwinkle plants were maintained in a greenhouse environment. To confirm the presence of HLB infection in these periwinkle plants, polymerase chain reaction (PCR) analysis was performed using the OI1/OI2c primers (Bové, 2006; Yang et al., 2024).

Healthy periwinkle seeds were germinated in a greenhouse at the Fujian Academy of Agricultural Sciences, Fuzhou, China, under controlled conditions, with daytime temperatures set at 28 ± 3 °C and nighttime temperatures at 20 ± 3 °C, and relative humidity maintained between 45 and 80%. The plants were grown in loamy soil, with regular watering and nutrient supply to ensure steady growth. Five-leaf seedlings were transplanted into larger pots and kept under the same conditions.

Nine healthy periwinkle plants were grafted with healthy periwinkle scions to form the healthy (H) group. For the infected group (D), 18 healthy periwinkle plants were grafted with branches from HLB-infected periwinkles. The nine plants showing typical HLB symptoms were randomly selected for this group. The experimental design followed a randomized complete block design, consisting of nine infected (D) and nine healthy (H) plants, with three replications (three plants per replication) under greenhouse conditions.

**Figure 1:**
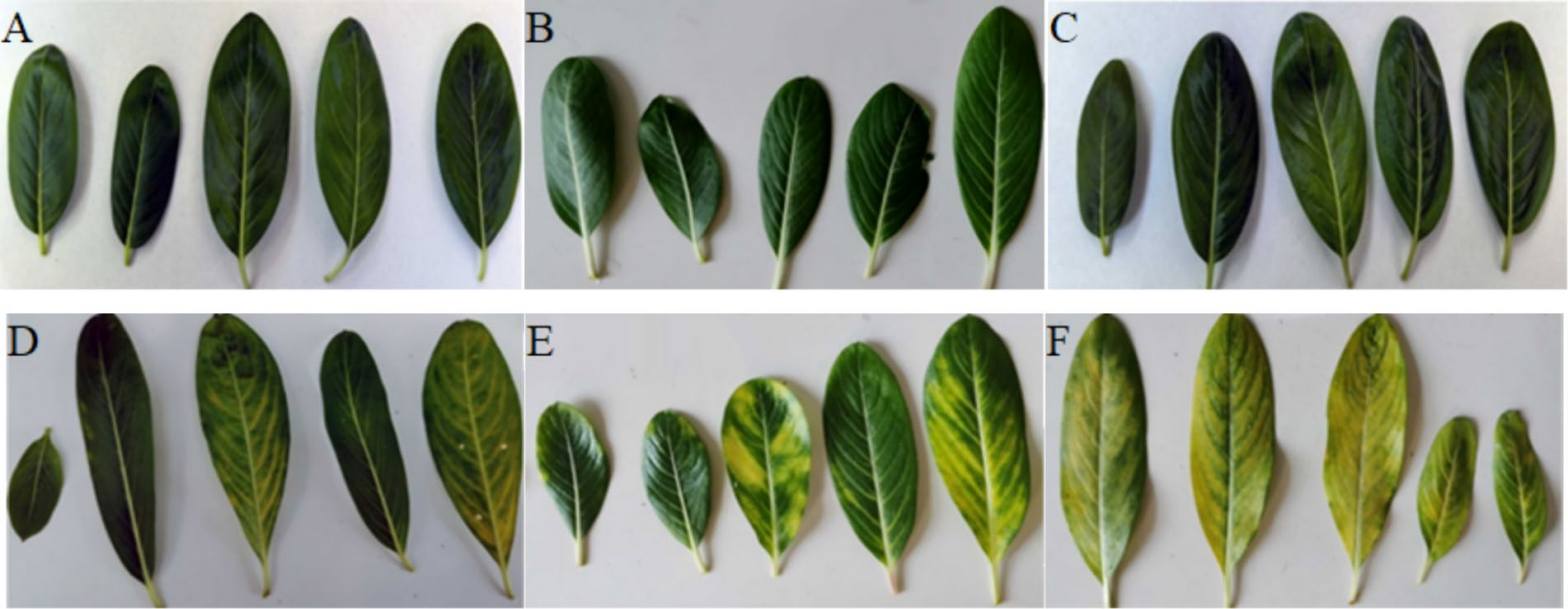
Three sampling stages of the periwinkle leaves: **(A)** Healthy leaves at day 30 (H1). **(B)** Healthy leaves at day 60 (H2). **(C)** Healthy leaves at day 90 (H3). **(D)** Diseased leaves at day 30 (D1). **(E)** Diseased leaves at day 60 (D2). **(F)** Diseased leaves at day 90 (D3).

Leaves from each of the plants were collected at three harvest times: 30 days (D1 and H1), 60 days (D2 and H2), and 90 days (D3 and H3) post-infection (Figure 1; Supplementary Table 1). Leaves of the same age, size, and shape were selected at each harvest time. They were rinsed with tap water, soaked in 75% ethanol for 1 min three times, and then washed two times with sterile distilled water. The leaves were also treated with 4% sodium hypochlorite solution for 4 min. The leaves were then rinsed several times with sterile distilled water. To confirm the sterility of the surface, 100 μL of the final rinse solution was plated onto LB agar plates and incubated at 28 °C for 2 days. Following this sterilization procedure, the samples were immediately frozen in liquid nitrogen.

### 2.2 Endophytic bacteria identification

Representative pooled samples from identical biological replicates were used for analysis. Total genomic DNA was extracted using the PowerSoil® DNA Isolation Kit (MO BIO, Carlsbad, California, USA), following the manufacturer’s protocol. The V3–V4 hypervariable regions of the 16S rRNA gene were amplified using KOD FX Neo DNA polymerase (TOYOBO, Osaka, Japan) with the primers 335F (5′-CADACTCCTACGGGAGGC-3′) and 769R (5′-ATCCTGTTTGMTMCCCVCRC-3′). PCR amplification conditions included an initial denaturation at 95 °C for 5 min, followed by 35 cycles of 95 °C for 30 s, 57 °C for 30 s, and 72 °C for 40 s, with a final extension step at 72 °C for 7 min. The reactions were held at 4 °C upon completion. Sequencing of the PCR products was conducted on the Illumina HiSeq 2,500 platform (Biomarker Technologies Corporation, Beijing, China).

### 2.3 Statistical analysis

Raw paired-end reads were first merged and quality-filtered using FLASH v1.2.7 and Trimmomatic v0.33 to generate clean tags. Chimeric sequences were then identified and eliminated by comparing them to a reference database using UCHIME version 4.2, resulting in high-quality tags. Operational taxonomic units (OTUs) were clustered based on 97% sequence similarity, and taxonomic classification was performed by aligning sequences with the SILVA database using QIIME version 1.9.1.

To assess biodiversity, both alpha and beta diversity metrics were calculated. The Shannon and abundance-based coverage estimator (ACE) indices were computed in QIIME, and statistical analysis was carried out in R (version 4.4.2) using the ggpubr and ggplot2 libraries. Rarefaction curves were generated using Mothur software (v1.22.2). To determine beta diversity, principal coordinate analysis (PCoA) of Bray–Curtis distance was conducted in QIIME and visualized using R. Functional profiling of microbial communities was predicted using PICRUSt2 with Kyoto Encyclopedia of Genes and Genomes (KEGG) pathway annotation, performed on the BMKCloud platform.^1^ Statistical comparisons were performed using parametric tests (Student’s *t*-test or ANOVA, two-sided, 95% CI) for data that met normality assumptions (assessed via Minitab 17). For data that did not meet normality, non-parametric tests (Wilcoxon or Kruskal-Wallis H test) were employed.

## 3 Results

### 3.1 Confirmation of HLB infection and results of OTU analysis

**Figure 2:**
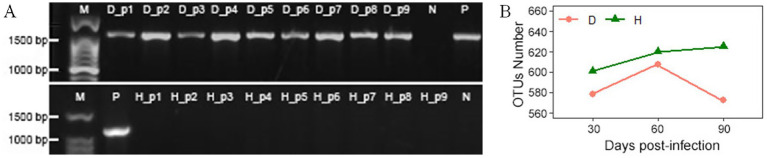
**(A)** Electrophoresis results of PCR products from periwinkle leaves using the OI1/OI2c primer pair on a 1.2% agarose gel. M: molecular weight marker; D_p1 to D_p9: HLB-affected periwinkle plants; H_p1 to H_p9: healthy periwinkle plants; P: positive control (HLB-confirmed plant); N: negative control (water). **(B)** OTU counts of endophytic bacteria in leaves of healthy (H) and infected (D) plants at 30, 60, and 90 days post-infection.

The presence of huanglongbing (HLB) infection was validated via PCR amplification using the OI1/OI2c primer pair, targeting a 1,167 bp fragment. Electrophoresis of the PCR products showed the expected amplicon in HLB-infected periwinkle plants (D_p1–D_p9) and the positive control (P), but no amplification was observed in healthy plants (H_p1–H_p9) or the negative control (N) (Figure 2A).

Sequencing generated 7,201,857 raw paired-end reads across all samples. After quality-filtering, merging, and chimera removal, 5,710,124 clean tags were obtained (average: 317,229 per sample; average length: 400 bp; Q20 > 95.7%). They were clustered into 664 OTUs with 97% similarity. The sample notations are described in Supplementary Table 1.

**Figure 3:**
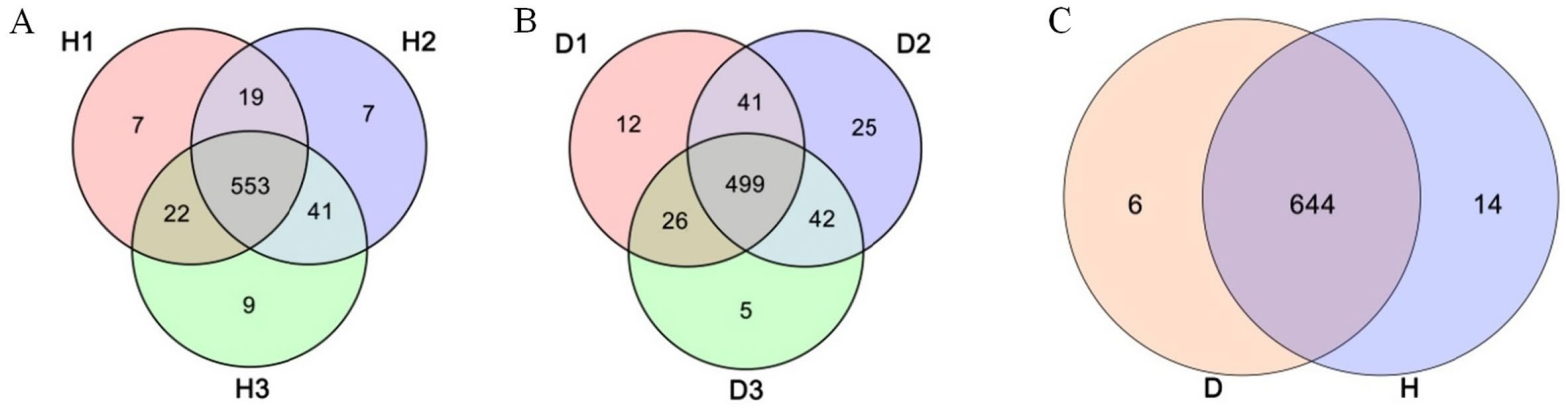
Venn diagram of OTUs. **(A)** Leaves of healthy plants at 30 (H1), 60 (H2), and 90 (H3) days post-infection. **(B)** Leaves of diseased plant at 30 (D1), 60 (D2), and 90 (D3) days post-infection. **(C)** Groups H (healthy plants) and D (diseased plants).

This study has compared the endophytic bacterial communities between healthy (H) and diseased (D) periwinkle plants at 30, 60, and 90 days post-infection. Group D consistently showed fewer OTUs than group H at each time stage (Figure 2B), with H1, H2, and H3 containing 7, 7, and 9 unique OTUs, respectively (Figure 3A). Infected plants (D1-D3) shared 499 common OTUs, while maintaining 12, 25, and 5 unique OTUs at the respective time points (Figure 3B). Overall, groups H and D shared 644 common OTUs, with healthy plants exhibiting greater unique OTU diversity than infected plants (Figure 3C).

### 3.2 Alpha diversity of endophytic bacteria

**Figure 4:**
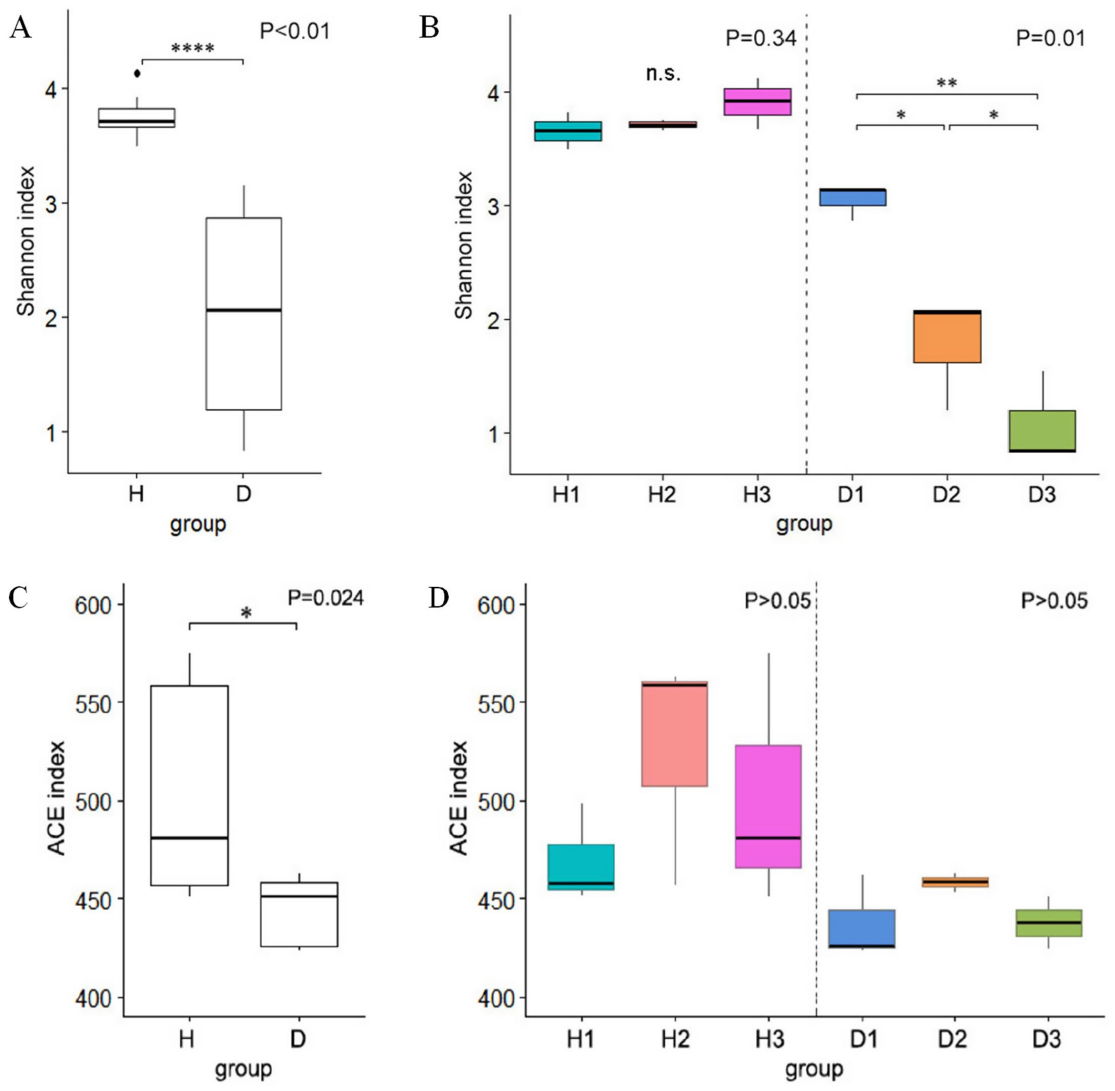
Boxplots of alpha diversity using the Shannon index: **(A)** comparison between healthy (H) and diseased (D) groups; **(B)** comparison at 30 (H1 and D1), 60 (H2 and D2), and 90 (H3 and D3) days post-infection. Boxplots of alpha diversity using the ACE index: **(C)** healthy vs. diseased groups; **(D)** comparison at the three time points.

Alpha diversity analysis demonstrated significantly higher bacterial diversity in healthy plants (group H; mean Shannon index = 3.76) than in HLB-affected plants (group D; mean Shannon index = 1.97, *p <* 0.01, Student’s *t*-test; Figure 4A). The rarefaction curves indicated saturation of the sequencing depth (Supplementary Figure 1). Group H maintained stable diversity across the time points (30 days post-infection, 3.66; 60 days post-infection, 3.71; 90 days post-infection, 3.91; *p* > 0.05, ANOVA), whereas group D exhibited a progressive decline in diversity, with significant decreases at D2 and D3 relative to D1 (*p <* 0.05, ANOVA, Figure 4B). ACE index analysis revealed significantly reduced bacterial richness in group D when compared to group H (*p <* 0.01, Wilcoxon test, Figure 4C); however, neither group showed temporal variation in richness (p > 0.05, Kruskal-Wallis H test, Figure 4D).

### 3.3 Beta diversity of endophytic bacteria

**Figure 5:**
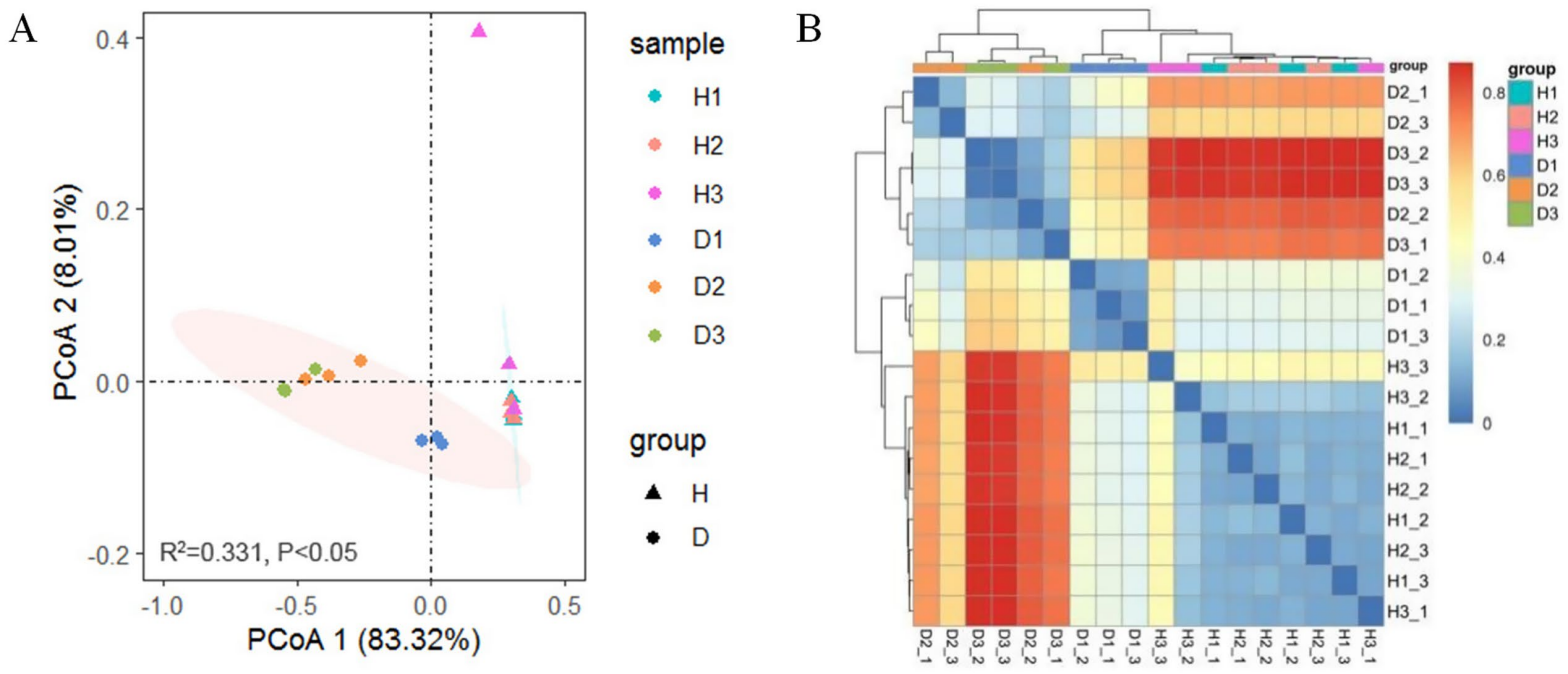
Beta diversity: **(A)** PCoA based on Bray-Curtis distance for OTUs from groups H and D at 30 (H1 and D1), 60 (H2 and D2), and 90 (H3 and D3) days post-infection (PerMANOVA); **(B)** Heatmap of Bray-Curtis distance illustrating bacterial community composition.

PCoA of Bray–Curtis distances revealed a clear separation in bacterial community composition between groups H and D (Figure 5A). Permutational multivariate analysis of variance (PerMANOVA) indicated that the bacterial community structure of periwinkle leaves among the clusters of the six groups (R^2^ = 0.331, *p <* 0.05, two-sided, 95% CI) differed significantly. Both groups exhibited relatively clustered intragroup structures across all time points (30, 60, and 90 days post-infection), except for one outlier, H3_3. In group H, the samples showed small within-group differences at all collection times. In group D, the bacterial community showed a greater difference in D1 than in D2 and D3, and the similarity of the bacterial community composition of D2 and D3 was relatively closer than that of D1. These patterns were confirmed by heatmap analysis (Figure 5B), which additionally demonstrated a close similarity between samples D2_2 and D3_1, as well as a relatively stable community structure in group H across the three time points. Together, these results indicated significant differences between endophytic bacterial communities of groups H and D, with infection duration notably affecting community composition in group D.

### 3.4 Taxonomic composition of endophytic bacteria

**Figure 6:**
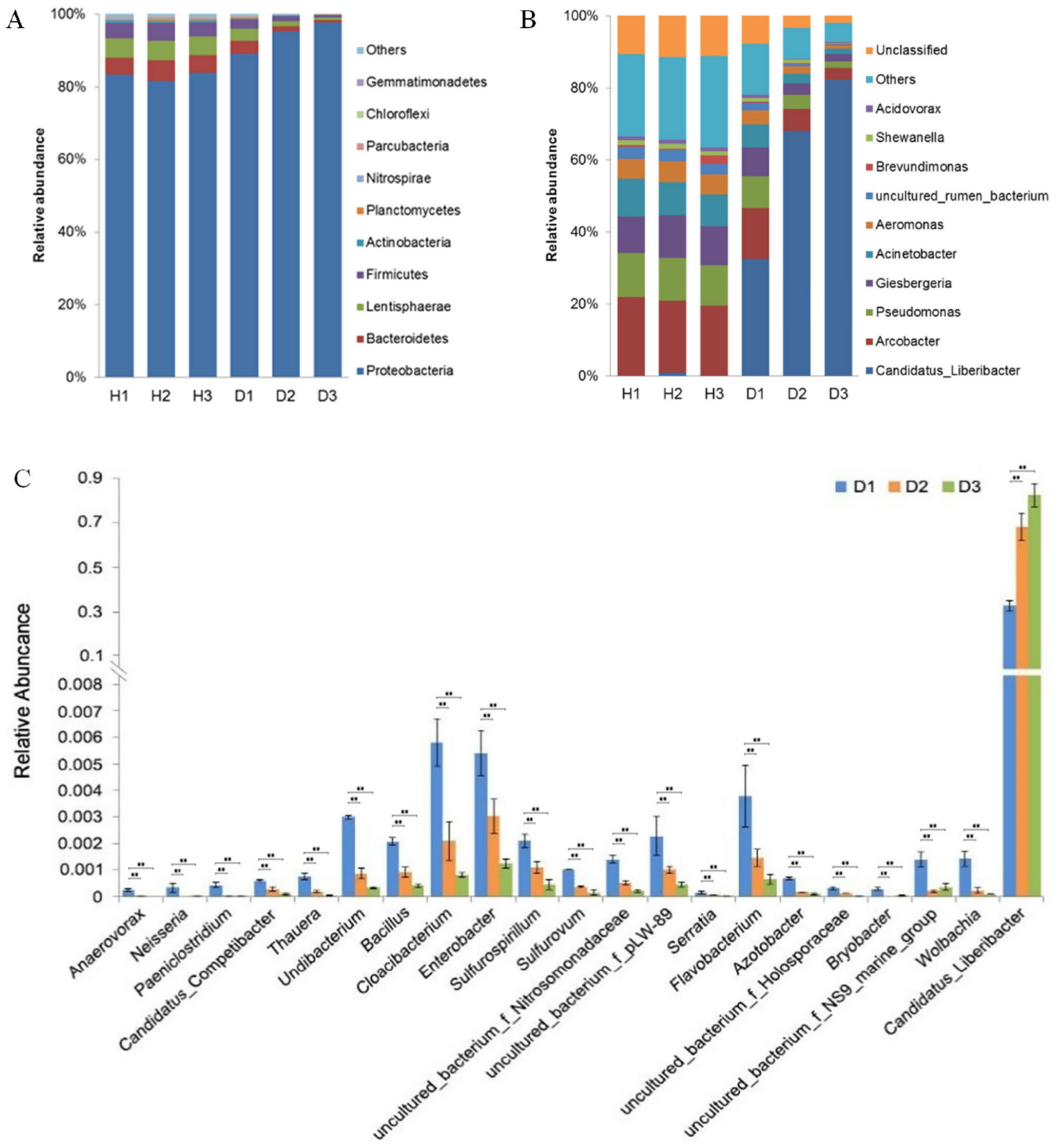
**(A)** Top 10 phyla in groups H (healthy) and D (diseased) at 30 (H1 and D1), 60 (H2 and D2), and 90 (H3 and D3) days post-infection. **(B)** Top 10 genera in groups H and D at the same time points. **(C)** Most variable genera and *Candidatus Liberibacter* in group D at 30 (H1 and D1), 60 (H2 and D2), and 90 (H3 and D3) days post-infection (**p < 0.01, ANOVA).

The OTU annotation identified 24 bacterial phyla and 300 genera in periwinkle leaf samples. (Supplementary Table 2). At the phylum level, Proteobacteria, Bacteroidetes, Lentisphaerae, and Firmicutes were the dominant groups (Figure 6A). In healthy plants, the relative abundance of these phyla remained consistent, while in diseased plants, shifts were observed. The proportion of Proteobacteria increased from 89.11% at 30 days post-infection to 97% by 90 days, while Bacteroidetes, Lentisphaerae, and Firmicutes declined progressively in group D.

Genus-level analysis revealed *Arcobacter*, *Pseudomonas*, and *Giesbergeria* were the most abundant taxa in healthy plants (Figure 6B); the outlier H3_3 was removed. In contrast, the plants in group D showed exponential growth of *Candidatus Liberibacter* (exceeding 80% relative abundance by Day 90), which coincided with a significant decline in other genera. The relative abundance of *Arcobacter* and *Pseudomonas,* the second (14.17%) and third (8.88%) most dominant genera in group D, respectively, declined significantly to 3.15 and 1.96% during HLB infection. ANOVA identified 20 genera with the lowest *p*-values (*p <* 0.05), including *Candidatus Liberibacter* (*p <* 0.05) (Figure 6C). The Benjamini-Hochberg false discovery rate analysis was used for testing. In group D, the most affected genera were *Anaerovorax*, *Neisseria*, and *Paeniclostridium*, whose relative abundance decreased to approximately 0% and also remained very low from Day 60 onward. The relative abundance of genera such as *Candidatus*, *Competibacter*, *Thauera*, *Undibacterium*, and *Bacillus* gradually decreased over time because of the infection.

### 3.5 Potential function of endophytic bacteria

**Figure 7:**
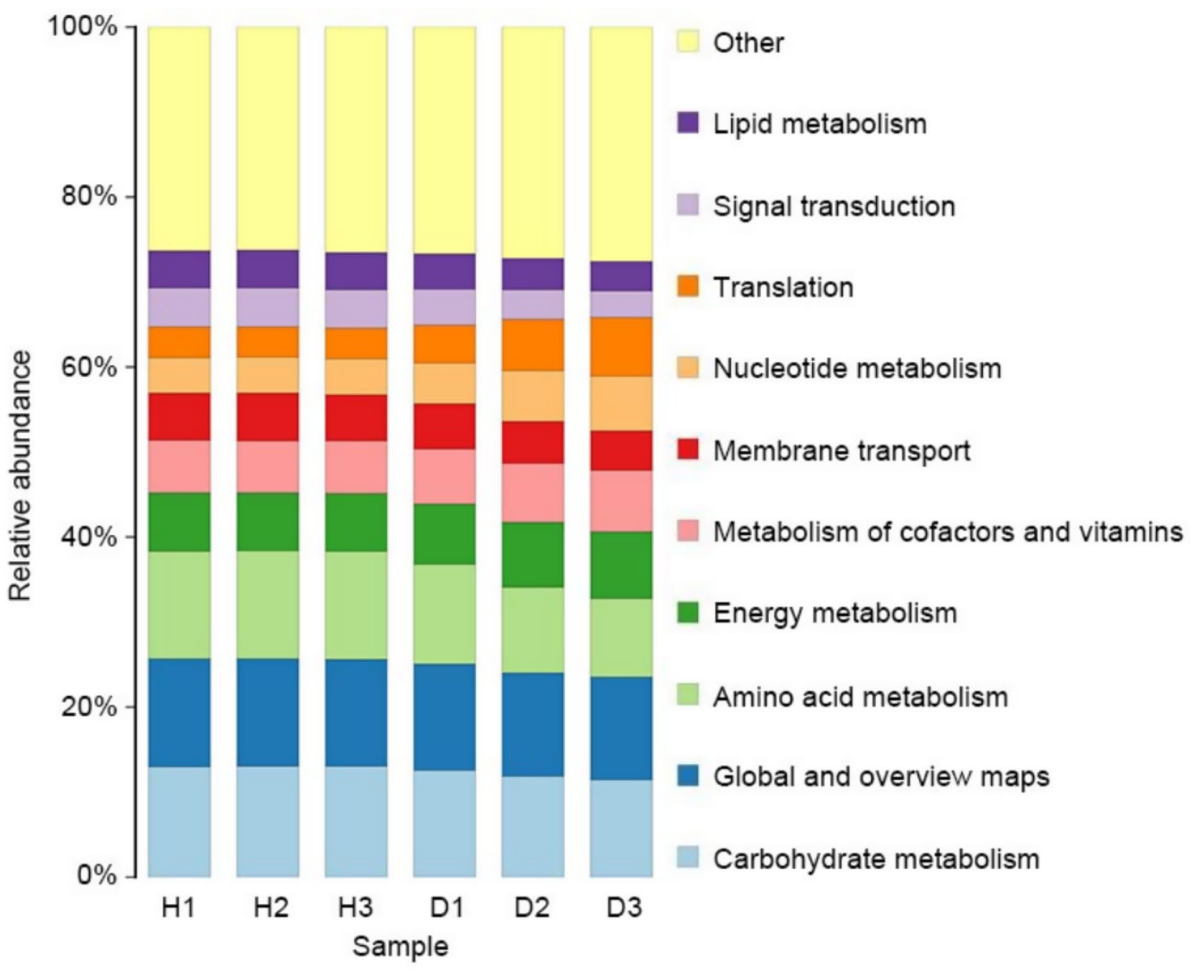
Endophytic bacterial community comparison of KEGG level 2 pathways for bacteria in groups H (healthy) and D (diseased) at 30 (H1 and D1), 60 (H2 and D2), and 90 (H3 and D3) days after infection.

The potential functions of the endophytic bacterial communities were predicted using the KEGG database through PICRUSt2, excluding the outlier H3_3 (Figure 7). Seven bacterial KEGG pathways related to metabolism were identified: carbohydrate metabolism, global overview, amino acid metabolism, energy metabolism, metabolism of cofactors and vitamins, nucleotide metabolism, and lipid metabolism. Two pathways, membrane transport and signal transduction, were identified in the environmental information processing cluster. A translation pathway was detected in the genetic information processing cluster. Metabolic pathways had higher relative abundance than other clusters. In group H, the abundance of the 10 bacterial KEGG pathways remained stable across time points (*p* > 0.05), whereas in group D, all 10 pathways exhibited significant changes over time due to HLB infection (*p <* 0.01). Specifically, the relative abundance of translation, nucleotide metabolism, energy metabolism, and metabolism of cofactors and vitamins increased in group D, while the other six pathways showed a significant decrease.

## 4 Discussion

Endophytic bacteria reside in various plant tissues and play a central role in the health of plants through involvement in the acquisition of nutrients, stress tolerance, and pathogen defense (Harrison and Griffin, 2020; Dominguez et al., 2023). Their community structure may be affected by a wide range of factors, including plant species, tissue type, developmental stage, environmental conditions, and pathogen infection (Dominguez et al., 2023; Liang et al., 2024). For this study, we used periwinkle as a model plant under controlled greenhouse conditions to monitor temporal dynamics of endophytic bacterial communities following inoculation with *Candidatus Liberibacter*, the pathogen that causes citrus huanglongbing (HLB).

### 4.1 Community composition and diversity of endophytic bacteria in response to HLB infection

Our results highlighted a significant impact of HLB infection on the leaf endophytic bacterial diversity and composition. Bacterial richness and diversity in the healthy periwinkle remained relatively stable throughout the 90-day duration, suggesting a healthy and stable endophytic community. Predominant genera such as *Arcobacter*, *Pseudomonas*, and *Giesbergeria* occurred repeatedly, in accordance with previous reports of their prevalence in non-infected periwinkle and citrus trees (Trivedi et al., 2010; Wang et al., 2010; Li et al., 2012). However, the HLB-infected plants exhibited a significant decline in bacterial diversity over time, with significant differences observed at 60 and 90 days post-infection (Figure 4B). Even overall richness (ACE index) was reduced in the infected cohort (Figure 4C), but it did not significantly differ over time. The findings are in line with earlier studies indicating that pathogen invasion can disrupt microbial equilibrium and render the community simpler (Xiong et al., 2017; Liu et al., 2020).

At the genus level, *Candidatus Liberibacter* rapidly dominated within infected hosts, with relative abundance exceeding 80% at 90 dpi. This increase corresponded with a substantial reduction in other key genera, including *Arcobacter*, *Pseudomonas*, *Anaerovorax*, *Neisseria*, and *Paeniclostridium*—suggesting a suppressive effect of the pathogen on native endophytes (Wang et al., 2010; Li et al., 2012). Low levels of *Candidatus Liberibacter* were also detected in some healthy plants, possibly due to the presence of non-pathogenic species such as *Ca. L. brunswickensis* (Thapa et al., 2020; Mishra and Ghanim, 2022).

### 4.2 Dynamic changes of endophytic bacterial composition in response to HLB infection

In this dynamic study, the endophytic bacterial community of healthy periwinkle leaves did not change significantly over time, indicating that environmental impacts were minimal. Thus, the HLB bacteria played a major role in the changes in the endophytic bacteria over time in the diseased group. A large population of *Candidatus Liberibacter* cells was detected in infected samples in this study, which is consistent with previous studies that verified periwinkle as a good host for HLB studies (Villechanoux et al., 1992; Sagaram et al., 2009). *Candidatus Liberibacter* became the dominant genus within a short duration following HLB infection, explaining the rapid development of the HLB symptoms in periwinkle plants. Most bacterial genera showed an inverse relationship with the relative abundance of *Candidatus Liberibacter*, and no genus demonstrated a consistent positive correlation with the pathogen over time. One limitation of this study is the relatively small sample size, which may have reduced the sensitivity of detecting the shift in low-abundance taxa. Additionally, comparisons with field-based studies are inherently limited due to differences in host species and the use of controlled greenhouse conditions, which do not fully replicate natural environments. Despite these constraints, the controlled setting and standardized inoculation method used herein minimized environmental variability and uncertainty about infection time. This experimental design enhanced the reproducibility and reliability of observation and offered a solid foundation for the investigation of HLB-induced changes in plant-associated microbiota.

Endophytes confer protection to plants through pathogen inhibition (Xu et al., 2018). The assembly of endophytic bacterial communities is likely influenced by both plant–pathogen and microbe–microbe interactions (Xu et al., 2018). Endophytes can protect plants through various mechanisms such as direct antagonism (antibiosis) or indirect interactions (induced resistance) (Compant et al., 2020; Koul et al., 2022). The biocontrol strains of *Pseudomonas*, which are found in soil, rhizosphere, and plant tissues, can protect plants from pathogenic infection through niche competition, antibiotic production, and induction of systemic resistance in plants (Yan et al., 2008; Chen X. et al., 2018). *Pseudomonas putida* W619 produced high levels of indole-3-acetic acid and can metabolize phosphonoacetic acid and phenylacetic acid, 4-aminobutyrate to improve plant growth (Taghavi et al., 2009). As an antagonistic endophyte, *Pseudomonas piscium* inhibits the growth and virulence of *Fusarium graminearum* by manipulating histone modifications using phenazine-1-carboxamide (Chen et al., 2018). *Pseudomonas* strains enhance nitrogen fixation, thereby improving disease resistance in rice (Yan et al., 2008). *Bacillus,* found in the rhizosphere and different plant tissues (leaves, roots, and stems), has antagonistic effects on a wide range of plant pathogens and can promote plant growth (Miljaković et al., 2020). A previous study found that an endophytic *Bacillus* strain could reduce CLas by enhancing the resilience of the citrus microbiome (Munir et al., 2022). The endophytic bacteria, *Bacillus*, *Serratia*, *Azotobacter*, and *Pseudomonas*, have plant growth-promoting traits that recommend them as effective agents for disease management (Ei et al., 2024). Therefore, the control of endophytes, such as the cultivation of useful microbes or the application of antagonists, is considered an eco-friendly biocontrol practice for enhancing plant protection for crop production (Yang et al., 2025b).

### 4.3 Potential function of endophytic bacteria in response to HLB infection

PICRUSt2 analysis revealed that infection by HLB profoundly affected the metabolic functions of endophytic bacterial communities. KEGG pathways predicted to be enriched in infected plants were dominated by three functional categories: metabolic processes, environmental signal processing, and management of genetic information. It is necessary to maintain microbial diversity for ecosystem functions to be sustained (Gao et al., 2021). Endophytic bacteria have also been reported to promote plant defense mechanisms through the production of secondary metabolites that could act as physical or chemical barriers against microbes or could serve as signal molecules to induce plant immunity (Zhou et al., 2025). The induction of a disease such as HLB, however, disturbs the internal status of a plant and reduces microbial functional diversity (Gao et al., 2021; Yang et al., 2025a).

HLB infection is associated with a significant reduction in carbohydrate, amino acid, and lipid metabolism, indicating likely suppression of endophyte growth (Ma et al., 2022). Conversely, studies on *Amorphophallus konjac* infected with *Pectobacterium carotovorum* subsp. carotovorum showed induction of genes related to carbohydrate metabolism and transport (Yang et al., 2025a), indicating differences in pathogenic tactics. Notably, genes involved in protein translation were higher in HLB-diseased plants compared to healthy periwinkle plants, supporting the maintenance of vital cellular functions during stress and rendering the surviving endophytes more resilient (Yang et al., 2025a).

Studies have demonstrated that HLB causes phloem clogging and chloroplast ultrastructural damage, both of which disrupt nutrient transport within the plant. As a result of these stressors, numerous microbial processes such as those pertinent to nucleotide and energy metabolism, cofactor, and vitamin biosynthesis were elevated, highlighting adaptation to nutrient limitation. Ma et al. (2022) assert that HLB is an immune-activating pathogen, often inducing cell damage and death in host plants. These changes appear to benefit the HLB pathogen, limiting the growth of other beneficial microbes. The induced change in metabolic activity illustrates a general disruption in the function and balance of the plant’s native microbiome caused by HLB infection.

## 5 Conclusion

Huanglongbing bacteria demonstrated rapid growth in relative abundance, reflecting their dominance in the microbial community in periwinkle plants over a short duration. HLB infection introduced tremendous changes in the endophytic bacterial populations over time, and the majority of the bacterial populations presented a steady decrease in relative abundance. The decrease may result from alterations in the internal environment after infection. No genus revealed a positive correlation with the HLB bacterium during the entire study. A larger sample size in future studies can help solidify these results. In addition, metatranscriptomic analysis could contribute further insights into the functional role of endophytic bacteria in the plants. This research contributes to our understanding of the invasion processes of the HLB pathogen and its effects on the native endophytic microbiome. It provides a platform to explore new avenues in HLB pathology and potential chemical or biocontrol interventions.

## Data Availability

The original contributions presented in the study are included in the article/Supplementary material, further inquiries can be directed to the corresponding author. Raw sequence reads were uploaded at the Sequence Read Archive of the National Center for Biotechnology Information (accession ID: PRJNA1304744).

## Footnotes

^1^ www.biocloud.net
